# Association between depression and young-onset dementia in middle-aged women

**DOI:** 10.1186/s13195-024-01475-y

**Published:** 2024-06-26

**Authors:** Jung Eun Yoo, Dae Hyun Yoon, Eun Hyo Jin, Kyungdo Han, Su-Yeon Choi, Seung Ho Choi, Jung Ho Bae, Kyung-Il Park

**Affiliations:** 1 Department of Family Medicine, Seoul National University Hospital Healthcare System Gangnam Center, Seoul National University College of Medicine, 39F Gangnam Finance Center 152, Teheran-ro, Gangnam-gu, Seoul, 06236 South Korea; 2 Department of Psychiatry, Seoul National University Hospital Healthcare System Gangnam Center, 39F Gangnam Finance Center 152, Teheran-ro, Gangnam-gu, Seoul, 06236 South Korea; 3 Department of Internal Medicine, Seoul National University Healthcare System Gangnam Center, Seoul National University College of Medicine, 39F Gangnam Finance Center 152, Teheran-ro, Gangnam-gu, Seoul, 06236 South Korea; 4 Department of Statistics and Actuarial Science, Soongsil University, 369 Sangdo-ro, Dongjak- gu, Seoul, 06978 South Korea; 5 Department of Neurology, Seoul National University Hospital Healthcare System Gangnam Center, Seoul National University College of Medicine, 39F Gangnam Finance Center 152, Teheran-ro, Gangnam-gu, Seoul, 06236 South Korea

**Keywords:** Depression, Dementia, Menopause, Menarche, Young-onset dementia

## Abstract

**Background:**

Dementia is associated with older adults; however, it can also affect younger individuals, known as young-onset dementia (YOD), when diagnosed before the age of 65 years. We aimed to conduct a retrospective cohort study involving middle-aged women to investigate the association between premorbid depression and YOD development.

**Methods:**

We included 1.6 million women aged 40–60 years who underwent health checkups under the Korean National Health Insurance Service and investigated the association between depression and YOD.

**Results:**

Women with depression had a significantly higher risk of developing YOD than women without depression. Among premenopausal women, those with depression had a 2.67-fold increased risk, whereas postmenopausal women with depression had a 2.50-fold increased risk. Late age at menarche (> 16 years) and young age at menopause (< 40 years) was associated with an increased risk of YOD.

**Conclusions:**

Depression in middle-aged women is a significant risk factor for the development of YOD. Understanding the role of reproductive factors can aid in the development of targeted therapeutic interventions to prevent or delay YOD.

**Supplementary Information:**

The online version contains supplementary material available at 10.1186/s13195-024-01475-y.

## Background

Dementia is a neurodegenerative condition, which often associated with older adults (late-onset dementia [LOD] ≥ 65 years) [1]. Dementia can also affect younger individuals, known as young-onset dementia (YOD), when diagnosed before the age of 65 years [2]. A recent meta-analysis showed that the global prevalence of YOD was 119.0 per 100,000 people aged 30–64 years [3]. This condition gives rise to socioeconomic challenges, as individuals under 65 years old are typically engaged in work and family caregiving responsibilities. Moreover, the diagnosis is frequently delayed due to its lower prevalence and larger variety of etiologies [4], which could lead to missing timely opportunities for treatment to delay symptom onset and provide appropriate care [2].

Identifying modifiable and non-modifiable risk factors is pivotal to develop targeted interventions and enhance our comprehension of underlying mechanisms. The apolipoprotein E (APOE) ε4 allele is a significant genetic risk factor for LOD [5]; however, association with YOD remains unclear. A recent study showed that individuals with two APOE ε4 alleles had a higher association with YOD compared to those with no ε4 allele [1, 6]. Additionally, mutations in the amyloid precursor protein (*APP*), presenilin 1 (*PSEN1*), and presenilin 2 (*PSEN2*) genes have been identified in patients with an autosomal-dominant form of early-onset Alzheimer’s disease [7]. However, the genetic influence on sporadic YOD is considerably limited [8]. Previous research supports an association between dementia and the following potential risk factors: less education, hypertension, hearing impairment, smoking, obesity, depression, physical inactivity, diabetes, social isolation, excessive alcohol consumption, traumatic brain injury, and air pollution [9]. Until recently, limited evidence existed regarding modifiable risk factors specifically focused on YOD. However, a recent prospective cohort study utilizing data from the UK Biobank has revealed risk factors significantly associated with a higher risk of YOD, including lower formal education, lower socioeconomic status, alcohol use disorder, social isolation, vitamin D deficiency, high C-reactive protein levels, stroke, diabetes, heart disease, and depression [1].

Depression is associated with an increased risk of LOD and YOD [1, 9, 10]. While depression can occur at any stage of life, when classified according to onset, late-life depression (≥ 60 years) increases the risk of dementia by 2 to 5 times [11]. A large-scale cohort study demonstrated that depression during early adulthood (18–44 years) and middle age (45–59 years) was also associated with dementia, with hazard ratios (HRs) of 3.08 (95% CI, 2.64–3.58) and 2.95 (95% CI, 2.75–3.17), respectively [12]. Depression and dementia are more prevalent in women than in men [13–15], and depressive episodes tend to cluster in the menopause transition [16–18]. This may indicate a link to sex-specific biological mechanisms, including hormonal fluctuations, which could play a role in the development of these conditions.

We, therefore, aimed to conduct a retrospective cohort study involving 1.6 million middle-aged women to investigate the association between premorbid depression and YOD development. Furthermore, given the potential association between depression, dementia, and fluctuations in female sex hormone levels, we hoped to emphasize the impact of menopausal status and female reproductive factors on this association.

## Methods

### Data source and study setting

The National Health Insurance Service (NHIS) is a single insurer in Korea that provides mandatory universal comprehensive medical care to 97% of the population and an additional medical aid program to 3% of the population in the lowest-income bracket. The NHIS provides biennial health screening for all insured individuals [19]. The database contains qualifications (e.g., age, sex, income, region, and type of eligibility), claims (general information on specifications, consultation statements, diagnosis statements defined by the International Classification of Disease, 10th revision [ICD-10], and prescription statements), health screening (self-questionnaire on health behaviors, anthropometric measurements, and laboratory test results), and mortality. In addition, the NHIS provides cancer screening programs targeting stomach, liver, colorectal, breast, and cervical cancers for all individuals exceeding the cancer-specific target age. All Korean women over the age of 20 years are instructed to be screened for cervical cancer biennially and those over 40 years are screened for breast cancer biennially.

### Study population

**Fig. 1:**
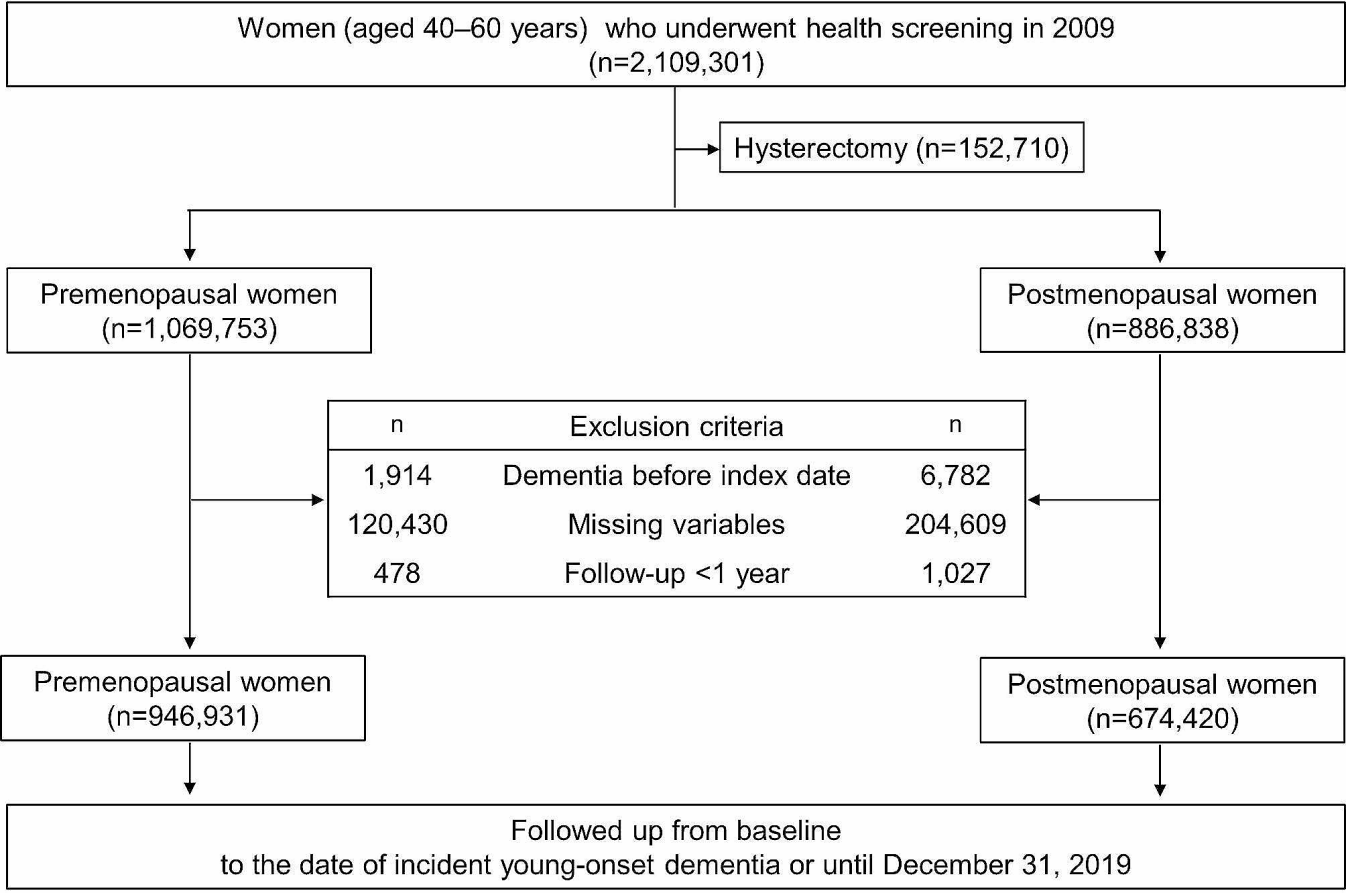
Flow chart of the study population

We initially identified 2,109,301 women (aged 40–60 years) who underwent both cardiovascular and breast/cervical cancer screening between 1 January 2009 and 31 December 2009. After excluding those who had a hysterectomy (*n* = 152,710), there were 1,069,753 and 886,838 eligible pre- and postmenopausal women, respectively. Among these, we excluded those (i) who were diagnosed with dementia before the health screening date (*n* = 1914 and *n* = 6782, respectively); (ii) who had at least one missing variable of interest (*n* = 120,430 and *n* = 204,609, respectively); and (iii) with follow-up of < 1 year (*n* = 478 and *n* = 1027, respectively). In total, 946,931 premenopausal and 674,420 postmenopausal women were included in the final analyses (Fig. 1).

This study was approved by the Institutional Review Board of Soognsil University (file no. SSU-202,007-HR-236-01). The review board waived the requirement for written informed consent because of the publicly available and anonymous data used for the analysis and the retrospective nature of the study.

### Depression and reproductive factors

Depression was defined as the presence of International Classification of Diseases (ICD)-10 codes F32 or F33. Information on reproductive factors including menopausal status was collected using a self-administered questionnaire during cervical and breast cancer screening details in supplementary figure. The recorded reproductive factors were as follows: age at menarche was categorized as ≤ 12 years, 13–14 years, 15–16 years (reference group), and ≥ 17 years, to be consistent with the distribution of age at menarche among Korean women; age at menopause was categorized as < 40 years, 40–44 years, 45–50 years, 51–54 years (reference group), and ≥ 55 years. The reference group for age at menarche and menopause was set as the group with the highest number of individuals within each age bracket (Table 1). Parity was categorized as 0, 1, or ≥ 2. Total lifetime breastfeeding history was categorized as never, < 6 months, 6–12 months, or ≥ 12 months. The duration of oral contraceptive use was categorized as never, < 12 months, or ≥ 12 months. History of hormone replacement therapy was dichotomized as yes or no.

**Table 1:** Baseline characteristics of the study population^a^

Characteristics	40–60-year-old women	
	Premenopausal women	Postmenopausal women
	*N* = 946,931	*N* = 674,420
Age at recruitment (years)	45.0 ± 4.0	54.8 ± 3.6
Smoking status		
Nonsmoker	899,439 (95.0)	646,036 (95.8)
Ex-smoker	15,167 (1.6)	8,129 (1.2)
Current smoker	32,325 (3.4)	20,255 (3.0)
Drinking		
Non	679,530 (71.8)	562,053 (83.3)
Mild	256,474 (27.1)	107,262 (15.9)
Heavy	10,927 (1.1)	5,105 (0.8)
Regular exercise	163,687 (17.3)	136,003 (20.2)
BMI level		
< 18.5	26,462 (2.8)	11,785 (1.8)
≥ 18.5, < 23	465,842 (49.2)	252,262 (37.4)
≥ 23, < 25	221,228 (23.4)	181,507 (26.9)
≥ 25, < 30	204,117 (21.5)	202,373 (30.0)
≥ 30	29,282 (3.1)	26,493 (3.9)
Low income	234,652 (24.8)	162,413 (24.1)
Diabetes mellitus	33,548 (3.5)	58,620 (8.7)
Hypertension	132,592 (14.0)	219,133 (32.5)
Dyslipidemia	101,959 (10.8)	201,405 (29.9)
Chronic kidney disease	39,628 (4.2)	35,781 (5.3)
Depression	31,336 (3.3)	39,885 (5.9)
Age at menarche		
≤ 12	41,863 (4.4)	9,849 (1.5)
13–14	297,848 (31.5)	109,848 (16.3)
15–16	433,982 (45.8)	287,705 (42.6)
> 16	173,238 (18.3)	267,018 (39.6)
Age at menopause	N/A	
< 40		8,688 (1.3)
40–44		30,946 (4.6)
45–50		192,025 (28.5)
51–54		390,605 (57.9)
≥ 55		52,156 (7.7)
Parity		
0	35,357 (3.7)	15,575 (2.3)
1	126,347 (13.3)	59,051 (8.8)
≥ 2	785,227 (83.0)	599,794 (88.9)
Duration of breast feeding		
No	172,324 (18.2)	62,816 (9.3)
< 6 month	229,361 (24.2)	62,580 (9.3)
≥ 6, < 12 month	249,258 (26.3)	145,539 (21.6)
≥ 12 month	295,988 (31.3)	403,485 (59.8)
Duration of oral contraceptive use		
No	821,407 (86.8)	568,036 (84.2)
< 12 month	92,184 (9.7)	67,737 (10.1)
≥ 12 month	33,340 (3.5)	38,647 (5.7)
Hormone replacement therapy (yes)	N/A	135,159 (20.0)

BMI: body mass index; N/A: not available

^a^Data are presented as mean ± standard deviation (SD) or n (%)

^b^Alcohol consumption was classified as none, mild (< 30 g of alcohol/day), or heavy (≥ 30 g of alcohol/day)

^c^Regular exercise was defined as high-intensity activity ≥ 3 times/week or moderate-intensity activity ≥ 5 times/week

### Definition of YOD

The endpoint of the study was newly diagnosed YOD occurring before 65 years of age. Dementia was defined as the prescription of acetylcholinesterase inhibitors (donepezil hydrochloride, rivastigmine, and galantamine) or an N-methyl-D-aspartate (NMDA) receptor antagonist (memantine), and, if the patient data included codes for Alzheimer’s disease (AD; ICD-10 F00 or G30), vascular dementia (VaD; ICD-10 F01), or other dementias (ICD-10 F02, F03, G23.1, or G31) [20, 21]. To file expense claims for acetylcholinesterase inhibitor or NMDA receptor antagonist (memantine) prescriptions for dementia treatment, Korean physicians need to document evidence of cognitive dysfunction according to the National Health Insurance Reimbursement criteria, including a Mini-Mental State Examination ≤ 26 and either a Clinical Dementia Rating ≥ 1 or a Global Deterioration Scale ≥ 3. The cohort was followed from baseline to the date of incident of YOD or until the end of the study period (31 December 2019), whichever occurred first.

### Covariates

We considered socioeconomic status, including income level, as a potential covariate. Information on current smoking status and alcohol consumption was obtained using a questionnaire. Study participants were classified as never, ex-, or current smokers. Alcohol consumption was classified as none, mild (< 30 g of alcohol/day), or heavy (≥ 30 g of alcohol/day). Regular exercise was defined as performing > 30 min of moderate physical activity at least five times per week or > 20 min of strenuous physical activity at least three times per week. Participants were categorized into five groups based on their body mass index (BMI, kg/m^2^) according to the Asia-Pacific criteria of the World Health Organization [22]. Comorbidities, such as hypertension, type 2 diabetes, dyslipidemia, chronic kidney disease, and depression, were based on claims data before the screening date and health screening results [23]. Hypertension was defined according to (i) the presence of at least one claim per year under ICD-10 codes I10-13 for the prescription of antihypertensive agents, or (ii) systolic blood pressure/diastolic blood pressure/diastolic blood pressure ≥ 140/90 mmHg. Type 2 diabetes was defined according to (i) the presence of at least one claim per year under ICD-10 codes E11-E14 codes for the prescription of a hypoglycemic medication or (ii) fasting glucose level ≥ 126 mg/dL. Dyslipidemia was defined as (i) the presence of at least one claim per year under ICD-10 code E78 for the prescription of a lipid-lowering agent, or (ii) total cholesterol ≥ 240 mg/dL. Chronic kidney disease (CKD) was defined as a glomerular filtration rate < 60 mL/min/1.73 m^2^ as estimated by the Modification of Diet in Renal Disease equation.

### Statistical analysis

Baseline characteristics are presented as means ± standard deviation or numbers and percentages. Continuous variables were compared using analysis of variance, whereas categorical variables were compared using the chi-squared test. Cox proportional hazards models were used to evaluate the association between depression and the incidence of dementia according to menopausal status. Model 1 was not adjusted; Model 2 was adjusted for age; Model 3 was adjusted for Model 2 + income, smoking, alcohol consumption, regular exercise, BMI, hypertension, type 2 diabetes, dyslipidemia, and CKD; and Model 4 was adjusted for Model 3 + age at menarche, age at menopause, parity, breastfeeding, oral contraceptive use, and hormone replacement therapy. We also conducted a stratified analysis by age at menarche in premenopausal women and age at menopause in postmenopausal women. Statistical analyses were performed using SAS version 9.4 (SAS Institute Inc., Cary, NC), and a *P* value < 0.05 was considered statistically significant.

## Results

### Baseline characteristics of the study population

The characteristics of the study population are summarized in Table 1. A total of 1,621,351 women aged 40–60 years participated in the study, with 946,931 women being in the premenopausal stage and 674,420 women in the postmenopausal stage. The prevalence of depression was 3.3% and 5.9% among pre-and postmenopausal women, respectively. Premenopausal women exhibited higher rates of current smoking, heavy alcohol consumption, and low incomes than postmenopausal women, who predominantly showed higher rate of regular exercise, diabetes mellitus, hypertension, dyslipidemia, chronic kidney disease, obesity (BMI ≥ 30 kg/m^2^), late age at menarche (> 16 years), multiple parities, history of breast feeding, and oral contraceptive use.

### Depression and risk of YOD (< 65 years)

A total of 9,197 cases of YOD were documented over an average follow-up period of 8.7 ± 1.5 years, with an average age at diagnosis of 53.4 ± 4.6 years. The incidence rate of YOD was 0.77 per 1,000 person-years in premenopausal women with depression and 3.24 per 1,000 person-years in postmenopausal women with depression. Premenopausal women with depression exhibited an elevated risk of YOD (adjusted HR [aHR], 2.67; 95% CI, 2.32–3.07) after adjusting for multiple variables, in comparison to the non-depression group (Table 2). Among postmenopausal women, depression was also associated with a higher risk of YOD (aHR 2.50; 95% CI, 2.34–2.67). Regarding the specific causes of dementia, depression showed a significant increase in the risk of AD in premenopausal women (aHR, 3.05; 95% CI, 2.59–3.59) and postmenopausal women (aHR, 2.63; 95% CI, 2.43–2.84) compared with those without depression. Compared with non-depressed groups, depression was also associated with a higher risk of VaD in both premenopausal and postmenopausal women (aHR 2.03; 95% CI, 1.46–2.82; aHR 1.85; 95% CI, 1.53–2.23), respectively.

**Table 2:** Incidence and risk of young-onset dementia diagnosed before the age of 65 years based on the status of depression and menopause

	Depression	No.	Event (*n*)	Person-years	IR^a^	HR (95% CI)			
						Model 1^b^	Model 2^c^	Model 3^d^	Model 4^e^
**Any dementia**									
Premenopausal women	No	915,595	1,972	8,505,861	0.23	1 (Ref.)	1 (Ref.)	1 (Ref.)	1 (Ref.)
	Yes	31,336	223	290,499	0.77	3.30 (2.88, 3.79)	2.88 (2.51, 3.31)	2.71 (2.54, 3.12)	2.67 (2.32, 3.07)
Postmenopausal women	No	634,535	5,998	5,056,422	1.19	1 (Ref.)	1 (Ref.)	1 (Ref.)	1 (Ref.)
	Yes	39,885	1,004	309,999	3.24	2.74 (2.57, 2.93)	2.63 (2.46, 2.81)	2.54 (2.37, 2.71)	2.50 (2.34, 2.67)
**Alzheimer’s dementia**									
Premenopausal women	No	915,595	1,254	8,505,861	0.15	1 (Ref.)	1 (Ref.)	1 (Ref.)	1 (Ref.)
	Yes	31,336	163	290,499	0.56	3.78 (3.22, 4.46)	3.26 (2.77, 3.84)	3.10 (2.63, 3.65)	3.05 (2.59, 3.59)
Postmenopausal women	No	634,535	4,260	5,056,422	0.84	1 (Ref.)	1 (Ref.)	1 (Ref.)	1 (Ref.)
	Yes	39,885	750	309,999	2.42	2.89 (2.67, 3.12)	2.77 (2.56, 2.99)	2.68 (2.48, 2.90)	2.63 (2.43, 2.85)
**Vascular dementia**									
Premenopausal women	No	915,595	457	8,505,861	0.05	1 (Ref.)	1 (Ref.)	1 (Ref.)	1 (Ref.)
	Yes	31,336	39	290,499	0.13	2.51 (1.81, 3.49)	2.28 (1.65, 3.17)	2.07 (1.49, 2.87)	2.03 (1.46, 2.82)
Postmenopausal women	No	634,535	985	5,056,422	0.19	1 (Ref.)	1 (Ref.)	1 (Ref.)	1 (Ref.)
	Yes	39,885	122	309,999	0.39	2.03 (1.68, 2.45)	1.97 (1.63, 2.37)	1.85 (1.53, 2.23)	1.85 (1.53, 2.23)

CI: confidence interval; HR: hazard ratio; IR: incidence rate; Ref.: reference

^a^Per 1,000 person-years

^b^Model 1 was unadjusted

^c^Model 2 was adjusted for age

^d^Model 3 was adjusted for age, smoking status, regular exercise, alcohol consumption, body mass index (BMI), hypertension, type 2 diabetes, dyslipidemia, and chronic kidney disease (CKD).

^e^Model 4 was adjusted for age, smoking status, regular exercise, alcohol consumption, BMI, hypertension, type 2 diabetes, dyslipidemia, CKD, age at menarche, age at menopause, parity, breast feeding, oral contraceptive use, and hormone replacement therapy

### Menarche age and risk of YOD among premenopausal women

Premenopausal women with depression exhibited a higher incidence of YOD than those without depression (Table 3). No significant trend was observed between the age at menarche and the risk of developing YOD in the depression group. However, in the non-depressed group, compared with women who experienced menarche at 15–16 years (reference group), those who experienced menarche at the ages of 13–14 years showed a slightly reduced risk of YOD (aHR 0.87; 95% CI, 0.77–0.98). In contrast, those who experienced menarche at the age of 16 years or older had a 36% elevated risk of all-cause YOD dementia and a 46% elevated risk of AD compared to the reference group (aHR 1.36; 95% CI, 1.22–1.50; aHR 1.46; 95% CI, 1.28–1.66). A significant inverse trend between age at menarche and YOD risk was noted, which was statistically significant in the non-depression group (*P* for trend < 0.0001).

**Table 3:** Association between age at menarche and risk of young-onset dementia based on depression status

	Age at menarche (years)	Premenopausal women without depression					Premenopausal women with depression				
		No.	Event	Person-years	IR^a^	Model 4^b^	No.	Event	Person-years	IR^a^	Model 4^b^
**Any dementia**											
	≤ 12	40,544	57	375,859	0.15	0.94 (0.72, 1.23)	1,319	6	12,209	0.49	0.96 (0.42, 2.20)
	13–14	288,936	418	2,683,112	0.16	0.87 (0.77, 0.98)	8,912	54	82,685	0.65	1.12 (0.80, 1.57)
	15–16	419,801	862	3,901,204	0.22	1 (Ref.)	14,181	92	131,482	0.70	1 (Ref.)
	> 16	166,314	635	1,545,685	0.41	1.36 (1.22, 1.50)	6,924	71	64,123	1.11	1.16 (0.85, 1.59)
	*P* for trend					< 0.0001					0.574
**Alzheimer’s dementia**											
	≤ 12	40,544	41	375,859	0.11	1.14 (0.83, 1.57)	1,319	4	12,209	0.33	0.89 (0.32, 2.43)
	13–14	288,936	255	2,683,112	0.10	0.89 (0.76, 1.03)	8,912	40	82,685	0.48	1.14 (0.77, 1.68)
	15–16	419,801	528	3,901,204	0.14	1 (Ref.)	14,181	69	131,482	0.52	1 (Ref.)
	> 16	166,314	430	1,545,685	0.28	1.46 (1.28, 1.66)	6,924	50	64,123	0.78	1.07 (0.74, 1.53)
	*P* for trend					< 0.0001					1
**Vascular dementia**											
	≤ 12	40,544	9	375,859	0.02	0.54 (0.28, 1.06)	1,319	1	12,209	0.08	0.97 (0.13, 7.38)
	13–14	288,936	113	2,683,112	0.04	0.89 (0.71, 1.13)	8,912	9	82,685	0.11	1.15 (0.50, 2.66)
	15–16	419,801	212	3,901,204	0.05	1 (Ref.)	14,181	14	131,482	0.11	1 (Ref.)
	> 16	166,314	123	1,545,685	0.08	1.15 (0.91, 1.44)	6,924	15	64,123	0.23	1.72 (0.83, 3.58)
	*P* for trend					0.016					0.279

IR: incidence rate; Ref.: reference

^a^Per 1,000 person-years

^b^Model 4 was adjusted for age, smoking status, regular exercise, alcohol consumption, body mass index, hypertension, type 2 diabetes, dyslipidemia, chronic kidney disease, age at menarche, age at menopause, parity, breast feeding, oral contraceptive use, and hormone replacement therapy

### Menopausal age and risk of YOD among postmenopausal women

In postmenopausal women, a younger age at menopause was associated with an elevated risk of all-cause YOD in both those with and without depression, as indicated in Table 4. Among women without depression, menopause before the age of 40 years, between 41 and 44 years, and between 45 and 50 years was associated with an increased risk of all-cause YOD by 67%, 59%, and 19%, respectively, in comparison to the women who underwent menopause between the ages of 51 and 54 years (reference group) (*P* for trend < 0.0001). Similarly, among women with depression, menopause occurring before the age of 40, 41–44, and 45–50 years was linked to an increased risk of all-cause YOD by 61%, 76%, and 24%, respectively, compared with the reference group (*P* for trend < 0.0001). Notably, early menopause (before 40 years of age) was associated with a 1.8-fold increased risk of AD among women without depression, and there was a dose-response relationship between younger age at menopause and AD (P for trend < 0.0001). Women with menopause and aged between 45 and 50 years had an associated higher risk of VaD (aHR 1.64, 95% CI 1.11–2.25), compared to the reference group.

**Table 4:** Association between age at menopause and risk of young-onset dementia based on depression status

	Age at menopause (years)	Postmenopausal women without depression					Postmenopausal women with depression				
		No.	Event	Person-years	IR^a^	Model 4^b^	No.	Event	Person-years	IR^a^	Model 4^b^
**Any dementia**											
	< 40	8,083	98	65,984	1.49	1.67 (1.36, 2.04)	605	21	4,763	4.41	1.62 (1.05, 2.50)
	40–44	28,964	320	240,290	1.33	1.59 (1.42, 1.79)	1,982	66	15,987	4.13	1.76 (1.36, 2.27)
	45–50	180,459	1,635	1,496,333	1.09	1.19 (1.12, 1.26)	11,566	296	93,283	3.17	1.24 (1.07, 1.42)
	51–54	367,975	3,447	2,943,068	1.17	1 (Ref.)	22,630	555	176,720	3.14	1 (Ref.)
	≥ 55	49,054	498	310,747	1.60	0.93 (0.85, 1.02)	3,102	66	19,247	3.43	0.76 (0.59, 0.98)
	*P* for Trend					< 0.0001					< 0.0001
**Alzheimer’s dementia**											
	< 40	8,083	74	65,984	1.12	1.83 (1.45, 2.31)	605	16	4,763	3.36	1.64 (0.99, 2.70)
	40–44	28,964	234	240,290	0.97	1.69 (1.48, 1.93)	1,982	44	15,987	2.75	1.56 (1.14, 2.12)
	45–50	180,459	1,184	1,496,333	0.79	1.24 (1.16, 1.33)	11,566	217	93,283	2.33	1.19 (1.01, 1.41)
	51–54	367,975	2,415	2,943,068	0.82	1 (Ref.)	22,630	425	176,720	2.40	1 (Ref.)
	≥ 55	49,054	353	310,747	1.14	0.95 (0.84, 1.06)	3,102	48	19,247	2.49	0.73 (0.54, 0.98)
	*P* for Trend					< 0.0001					0.0001
**Vascular dementia**											
	< 40	8,083	17	65,984	0.26	1.61 (0.99, 2.61)	605	3	4,763	0.63	2.19 (0.69, 6.98)
	40–44	28,964	43	240,290	0.18	1.19 (0.87, 1.63)	1,982	8	15,987	0.50	2.00 (0.95, 4.19)
	45–50	180,459	251	1,496,333	0.17	1.02 (0.88, 1.19)	11,566	42	93,283	0.45	1.64 (1.11, 2.45)
	51–54	367,975	599	2,943,068	0.20	1 (Ref.)	22,630	58	176,720	0.33	1 (Ref.)
	≥ 55	49,054	75	310,747	0.24	0.85 (0.66, 1.08)	3,102	11	19,247	0.57	1.28 (0.67, 2.44)
	*P* for Trend					0.027					0.031

IR: incidence rate; Ref.: reference

^a^Per 1,000 person-years

^b^Model 4 was adjusted for age, smoking status, regular exercise, alcohol consumption, body mass index, hypertension, type 2 diabetes, dyslipidemia, chronic kidney disease, age at menarche, age at menopause, parity, breast feeding, oral contraceptive use, and hormone replacement therapy

## Discussion

In this large cohort study of 1.6 million middle-aged women, depression was significantly associated with the risk of dementia before the age of 65 years, with a 2.7-fold increased risk among premenopausal women and a 2.5-fold increased risk among postmenopausal women compared with the non-depression group. Among premenopausal women without depression, late age at menarche (> 16 years) was associated with a 1.5-fold increased risk of YOD compared to the reference group. Among postmenopausal women, a younger age at menopause was associated with an increased risk of YOD; individuals with and without depression who experienced menopause before 40 years of age had a 67% and 62% increased risk of YOD, respectively.

A recent prospective cohort study showed that pre-existing depression is associated with a 3.25-fold higher risk of YOD [9]. Furthermore, a nationwide cohort study reported that depression increases the risk of YOD in men (HR 1.89, 95% CI 1.53–2.34) [24]. However, women have a higher likelihood of developing dementia over the course of their lives compared to men [15]. Recent meta-analyses have also revealed that women are more predisposed to develop YOD [25]. Nevertheless, to the best of our knowledge, there is a lack of evidence regarding the association between depression and YOD in women. This study represents the first exploration of the association between depression and YOD in middle-aged women, with a particular emphasis on reproductive factors, including menopausal status.

There has been considerable debate regarding whether depression acts as a risk factor for dementia or serves as a prodromal symptom [26]. This debate is fueled by the fact that depressed mood is one of the most common symptoms among dementia patients [11]. In particular, depression in older adults is often linked with mild cognitive impairment, which complicates the understanding of its precise association [14]. However, recent large-scale prospective cohort studies focusing on relatively younger age groups have indicated that depression occurring at least 10 years prior increases the risk of early-onset dementia [1, 24]. In line with this, we also investigated the risk of YOD in middle-aged women based on the presence of preceding depression, with an average follow-up period of 8.7 ± 1.5 years. Our findings suggest that depression indeed serves as a preceding risk factor for YOD.

The potential biological mechanisms connecting depression to dementia encompass several pathways. First, depression can affect neurovascular coupling by altering microvascular function, and these changes may contribute to the development and progression of dementia [27, 28]. Second, alterations in glucocorticoid levels due to depression activate the hypothalamic–pituitary–adrenal (HPA) axis, leading to hippocampal damage and chronic elevation of glucocorticoids, which contributes to hippocampal atrophy and cognitive deficits associated with dementia [11, 29]. Third, depression may involve increased β-amyloid production triggered by stress responses associated with dementia [29, 30]. Additionally, inflammatory modifications and deficiencies in nerve growth factors play pivotal roles in the pathophysiology of both depression and dementia [11].

Considering the significant hormonal changes occurring in middle-aged women, a subgroup analysis was conducted according to menopausal status and age at menarche or menopause. Our results showed that a later onset of menarche and an earlier onset of menopause were associated with an increased risk of dementia, consistent with prior studies [21, 31]. During the menopausal transition, women experience dramatic hormonal changes, which may impact both mood and cognitive decline [13, 18, 32, 33]. Neuroimaging studies have provided evidence that postmenopausal women exhibit lower gray matter density compared to perimenopausal women [34]. This is attributed to estrogen, a female sex hormone that may play a protective role against neural degeneration [35]. With aging, estrogen levels decline, leading to increased exposure to mitochondrial toxicity and beta-amyloid accumulation, increasing the risk of dementia [34].

According to the etiology of YOD, AD is indeed the most frequent cause, followed by VaD, frontotemporal dementia, and sequelae from traumatic brain injury [36]. When analyzing the results based on etiology, we divided the cohort into two categories: AD and VaD. Interestingly, the analysis revealed that the association between depression and YOD is more pronounced in AD than in VaD among women. Specifically, early menopause before the age of 40 was associated with a 1.6-fold increased risk of AD with depression and a 1.8-fold increased risk of AD without depression. However, this association of VaD is not statistically significant due to wide, overlapping confidence intervals. Therefore, the association between depression and YOD according to reproductive factors stems from AD rather than VaD. This result is consistent with well-known sex differences in AD dementia, which include the disproportionately higher prevalence and lifetime risk for developing AD dementia in women compared to men [37]. Moreover, our findings suggest that these differences extend to YOD, not only LOD.

According to the vascular-depression-dementia hypothesis, the relationship between depression and dementia is linked to cardiovascular diseases, particularly VaD [38]. The risk of dementia more than doubles in both men and women diagnosed with depression, with men experiencing a greater risk [12]. This is attributed to differences in health behaviors such as smoking and alcohol consumption, as well as the higher prevalence of accompanying cardiovascular risk factors like stroke, diabetes, and heart disease among men [11]. Our results indicated a lower HR for depression and VaD compared to AD due to our focus on women. Therefore, it is possible that the relative association between depression and VaD was underestimated.

This study had several limitations. First, several major YOD risk factors, such as APOE ε4 alleles; *APP*, *PSEN1*, and *PSEN2* genes; family history of dementia; history of traumatic brain injury; hearing loss; and education level could not be obtained [9]. Instead of education level, income level was included in the analyses because it is a substantial determinant of income. Second, we identified cases of dementia and depression solely based on ICD codes from nationwide health insurance claims data. This method carries an inherent risk of misdiagnosis, and such discrepancies may have resulted in inaccurate analyses. Subsequent studies could explore the possibility of supplementing these diagnoses with clinical examination results related to dementia, in addition to Diagnostic and Statistical Manual diagnoses. Third, treatment status for depression was not considered. It is important to acknowledge that factors such as the underlying causes of depression, severity of symptoms, and whether treatment was administered may impact the results.

## Conclusions

Depression in middle-aged women contributes to an increased risk of developing YOD, both before and after menopause. Female reproductive factors associated with hormonal changes may further exacerbate this association. These findings will provide a significant “treatment opportunity” to precisely target therapeutic interventions aimed at preventing or delaying YOD in women grappling with depression.

### Electronic supplementary material

Below is the link to the electronic supplementary material.

Supplementary Material 1

## Data Availability

No datasets were generated or analysed during the current study.

## Abbreviations

AD: Alzheimer’s disease
APP: Amyloid precursor protein
BMI: Body mass index
CKD: Chronic kidney disease
HRs: Hazard ratios
ICD: International Classification of Diseases
LOD: Late-onset dementia
NMDA: N-methyl-D-aspartate
NHIS: National Health Insurance Service
PSEN1: Presenilin 1
PSEN2: Presenilin 2
VaD: Vascular dementia
YOD: Young-onset dementia
