# Supplementary figures and images for: Association between depression and young-onset dementia in middle-aged women
Source: Alzheimers Res Ther. 2024 Jun 26;16:137. doi: 10.1186/s13195-024-01475-y (PMC11201295; doi:10.1186/s13195-024-01475-y)

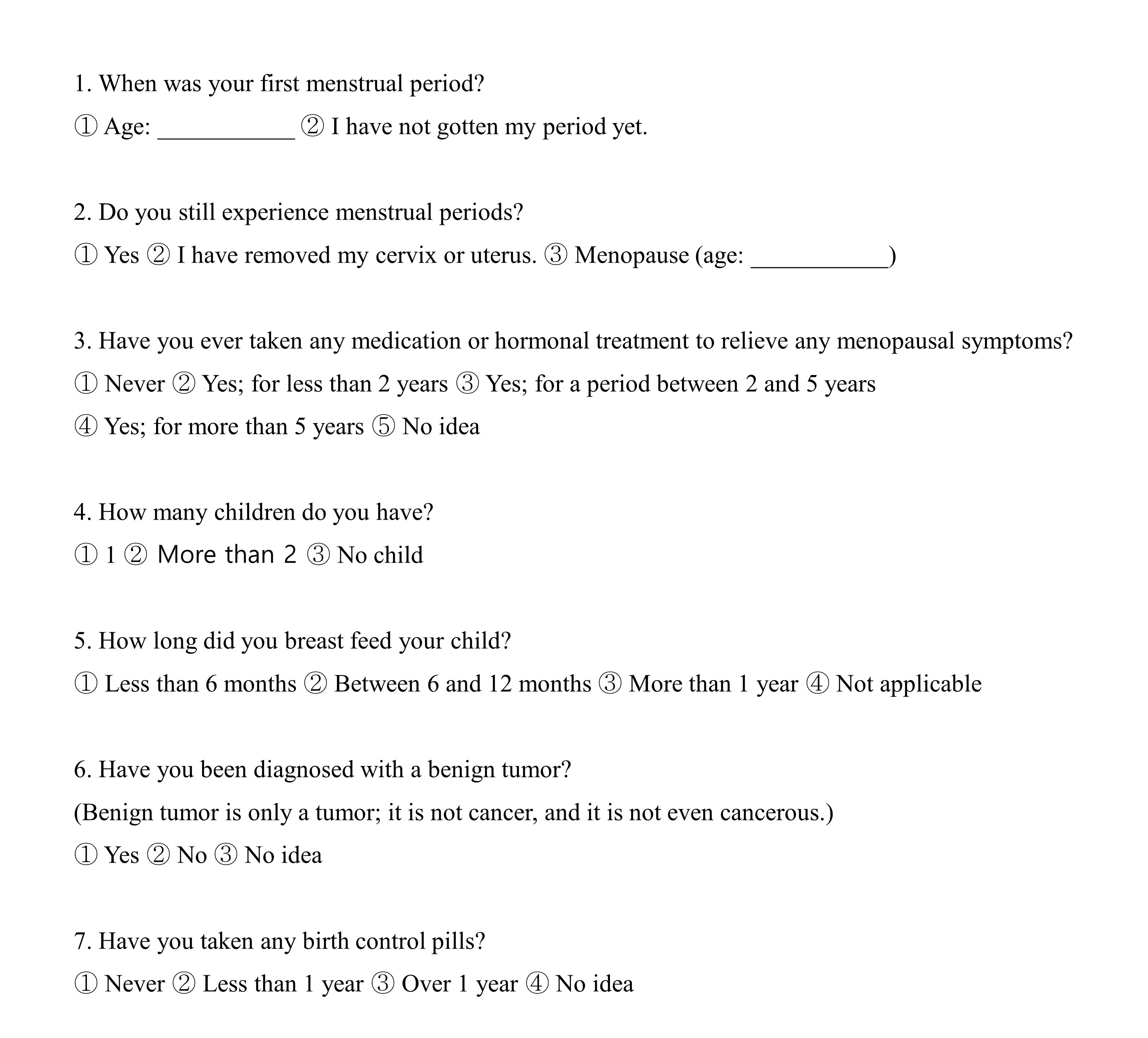

Supplement: Supplementary file 1 — Supplementary Material 1 [file 13195_2024_1475_MOESM1_ESM.jpg]
